# Improvisation and university students’ entrepreneurial intention in China: The roles of entrepreneurial self-efficacy and entrepreneurial policy support

**DOI:** 10.3389/fpsyg.2022.930682

**Published:** 2022-08-22

**Authors:** Runping Guo, Haobo Yin, Xingqun Lv

**Affiliations:** ^1^Department of Technological Economics and Management, School of Business and Management, Jilin University, Changchun, China; ^2^School of Entrepreneurship Education, Heilongjiang University, Harbin, China; ^3^Post-doctoral Research Station of Law, Heilongjiang University, Harbin, China

**Keywords:** improvisation, entrepreneurial self-efficacy, entrepreneurial intention, entrepreneurial policy support, China

## Abstract

In the VUCA era, determining how to deal with environmental uncertainty has become one of the core issues. Research shows that improvisation is an effective way to deal with rapid changes and to obtain unexpected opportunities in a complex and changeable environment. Improvisation, as a needed capability in the entrepreneurial process, can also provide key strategies to effectively deal with emergencies. Although previous studies have explored the outcomes of improvisation in the entrepreneurial field, this paper aims to investigate in depth whether and how improvisation affects entrepreneurial intention in China. A moderated mediation model was constructed and tested using data from 251 Chinese university students to explore the influence mechanism of improvisation on entrepreneurial intention by combining social cognitive theory and the entrepreneurial event model. The results of this empirical analysis found that improvisation has a positive effect on entrepreneurial intention and entrepreneurial self-efficacy. Entrepreneurial self-efficacy plays a fully mediating role in the relationship between improvisation and entrepreneurial intention. Moreover, entrepreneurial policy support has been found to significantly moderate the mediated relationship between improvisation and entrepreneurial intention by entrepreneurial self-efficacy. The findings suggest that individuals should cultivate improvisation capabilities and entrepreneurial self-efficacy to enhance their entrepreneurial intention. They also need to pay attention to the dynamics of entrepreneurial policies in China. This study contributes to the extant literature by providing deeper insight into the relationship between improvisation and entrepreneurial intention and also has important practical implications for promoting entrepreneurial intention formation in contexts with environmental uncertainty like China.

## Introduction

With the development of the economy, the expansion of universities and the negative impact of the Covid-19 pandemic, the employment situation of Chinese university graduates has become increasingly severe. University students are facing huge employment pressure. To alleviate this problem, the Chinese government encourages young people to start businesses and provides a large number of entrepreneurial preferential measures (Antoncic et al., 2015; He et al., 2019). However, entrepreneurship is a high-risk activity, and realistic factors, such as environmental uncertainty, resource scarcity, and information authenticity, make it difficult for university students to start a business (Fisher et al., 2021). University students’ entrepreneurship is an entrepreneurial process with the special group of college students and graduate students as the main body. University students are the main force of entrepreneurship in China, and it is vital to understand how university students generate entrepreneurial behavior to encourage entrepreneurship (Rodriguez-Gutierrez et al., 2020; Sheng and Chen, 2022). Prior research has examined the drivers of entrepreneurship by examining why individuals form entrepreneurial intention (Cai et al., 2020b; Wang et al., 2021a). In China, the largest transitional economy, the business, institutional and technological environment is highly distinctive and complex compared to mature market economies (Yu et al., 2018). Improvisation is a combination of intuition, creativity and bricolage driven by time pressure, which can improve aspects of adaptation and become an important way to cope with uncertainty and the complex environment (Malucelli et al., 2021). Consequently, improvisation seems to be the most reasonable way to understand the formation of entrepreneurial intention in China. However, in view of the extant literature, only Hmieleski and Corbett (2006) point out that improvisational individuals tend to seek entrepreneurial opportunities and generate entrepreneurial intention based on the mature economies. We know little about whether and how improvisation has an influence on entrepreneurial intention in China.

Moreover, the impact of improvisation on entrepreneurial intention is not autonomous, and it occurs through certain mediating variables. Based on social cognitive theory, Pfitzner-Eden (2016) indicates that individuals form beliefs about self-efficacy by interpreting information about their capabilities (Bandura, 1997). Similarly, entrepreneurial self-efficacy, a belief that individuals can effectively complete entrepreneurial activities and achieve success (Antoncic et al., 2015, 2021; Hsu et al., 2019; Edwards et al., 2022), may be affected by their capabilities, such as improvisation. The entrepreneurial event model posits that entrepreneurial intention stems from the feasibility of entrepreneurship, the perception of feasible future states related to starting a business successfully, which is influenced directly by entrepreneurial self-efficacy (Esfandiar et al., 2019; Cai et al., 2020b; Antoncic et al., 2021; Rakib et al., 2022). It can be seen that improvisation affects entrepreneurial intention through the bridge of entrepreneurial self-efficacy. Furthermore, potential entrepreneurs in China are faced with more significant unprecedented uncertainty than those in developed countries given that they are in a critical period of transforming its development mode, optimizing its economic structure and transforming its growth drivers at this stage (Yu et al., 2018). To cope with such environmental turbulence, individuals need to seize fleeting entrepreneurial opportunities. The policy orientation boosts entrepreneurial behavior and guides national economic development. Therefore, the entrepreneurial policy support has critical moderating effects on individual entrepreneurial choices.

Numerous indicators show that over the past three decades, emerging markets have become increasingly important in the global economy (Yu et al., 2018, 2020; Grover Goswami et al., 2019). As the world’s largest emerging economy, China’s entrepreneurial environment is full of uncertainty and unpredictability (Liu and Almor, 2016). This means that huge changes in market demand and rapid technological innovation have made environmental uncertainty a key feature that must be considered in entrepreneurial activities. Improvisation can help individuals effectively address the challenges posed by such environmental uncertainties (Best and Gooderham, 2015). Thus, compared with U.S. or European markets, improvisation research is more meaningful in the Chinese context. In addition, due to the particularity of the Chinese system, the government’s policy orientation has a substantial contingent impact on enterprises. Therefore, conducting this research in the context of China has important significance.

By integrating social cognitive theory and the entrepreneurial event model, we explore the influence mechanism of improvisation on entrepreneurial intention by examining the mediating role of entrepreneurial self-efficacy using data from China, the largest transitional economy. This study contributes to the extant literature in the following ways. First, it provides empirical evidence for the direct impact of improvisation on entrepreneurial self-efficacy and entrepreneurial intention in China’s transition economy. Second, by exploring the mediating role of entrepreneurial self-efficacy, this paper opens the “black box” of the relationship between improvisation and entrepreneurial intention. Finally, it provides new insights into the relationship between entrepreneurial self-efficacy and entrepreneurial intention under high uncertainty by exploring the antecedent impact of improvisation and the moderating effect of the entrepreneurial policy support.

## Theoretical background and conceptual model

Weick (1998) first introduced ideas that could improve organizational improvisation through descriptions of jazz improvisation. Magni et al. (2018) propose that improvisation is a process that can lead to personal gains or risks, and individual improvisation expresses a conscious choice that abandons established procedures to deal with emergencies (Leybourne and Sadler-Smith, 2006). When facing new problems or opportunities, environmental uncertainty makes it difficult to plan or utilize trial and error. Heuristic thinking appears to be more efficient than systematic thinking. Improvisation seems to be one of the most important abilities that potential entrepreneurs need to have (Hmieleski and Corbett, 2006; Gojny-Zbierowska and Zbierowski, 2021). In summary, we draw on prior work to define improvisation as an individual’s ability to use existing resources to achieve goals innovatively and spontaneously under tremendous pressure.

In social cognitive theory, triadic reciprocal causation is used to interpret psychosocial functioning. According to triadic reciprocal causation, behavioral, cognitive and other personal and environmental factors are the determinants of mutual influence (Wood and Bandura, 1989; Rakib et al., 2022). Entrepreneurial behavior is affected by cognitive and personal factors, such as entrepreneurial self-efficacy and personal capabilities (Bandura, 1977; Leybourne and Sadler-Smith, 2006; Antoncic et al., 2015; Schunk and DiBenedetto, 2020; Cai et al., 2020b). Improvisation as a personal capability allows individuals to seek opportunities to realize entrepreneurial behavior. Entrepreneurial intention is one of the most effective indicators of entrepreneurial behavior, which is usually defined as one’s desire to start a business (Bae et al., 2014; Esfandiar et al., 2019; Douglas et al., 2021; Gojny-Zbierowska and Zbierowski, 2021). Therefore, improvisation is essential for generating entrepreneurial intention. Also, improvisation has an impact on entrepreneurial self-efficacy. Bandura (1997) proposes that individuals develop self-efficacy by interpreting information about their capabilities, such as mastery experiences, vicarious experiences, verbal persuasion and physiological and affective states, which are the authentic indicators of one’s capabilities (Pfitzner-Eden, 2016). Having a functional coping ability undoubtedly contributes to a sense of personal efficacy (Bandura, 1977). Hence, improvisation is conducive to overcoming difficulties in entrepreneurship and enhancing entrepreneurial self-efficacy.

Moreover, the entrepreneurial event model demonstrates that entrepreneurial self-efficacy can impact entrepreneurial intention through entrepreneurial perceived feasibility (Bullough et al., 2014; Huang et al., 2021). Combined with social cognitive theory, entrepreneurial self-efficacy can be used as a pathway to explain the relationship between improvisation and entrepreneurial intention. Finally, contingency theory suggests that individuals’ behavioral effects change under different situations (Harrison, 2018). Government policy, one of the critical environmental factors in entrepreneurship, has significant support and guidance effects (Wang et al., 2016). Thus, we introduce entrepreneurial policy support to explore the impact of cognition on individual behavior under different regional entrepreneurial policies.

**Figure 1:**
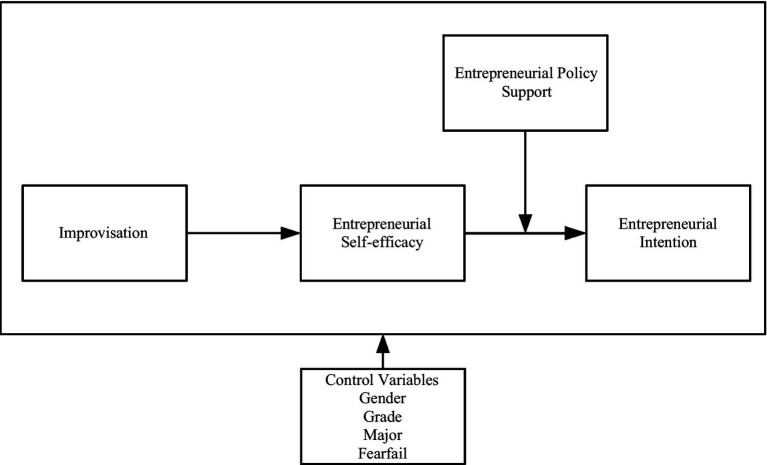
Research framework.

Applying social cognitive theory and the entrepreneurial event model, we construct a well-suited framework to examine how improvisation impacts entrepreneurial intention using data from China, the largest transitional economy characterized by turbulence and changes. First, as shown in Figure 1, we examine the effect of improvisation on entrepreneurial intention and entrepreneurial self-efficacy. Second, we examine the mediating role of entrepreneurial self-efficacy in the relationship between improvisation and entrepreneurial intention. Finally, we discuss the moderating role of entrepreneurial policy support (Figure 1).

## Hypothesis

### Improvisation and entrepreneurial intention

Improvisation consists of three dimensions: (1) creativity and bricolage, (2) the ability to function and excel under pressure-filled and stressful environments and (3) spontaneity and persistence (Hmieleski and Corbett, 2006). In general, improvisation focuses on using existing resources to spontaneously and creatively seize opportunities and achieve goals under time pressure and risk. According to social cognitive theory, improvisation is the core element of entrepreneurial motivation and the key to explaining entrepreneurial intention.

Specifically, as a dimension of improvisation, creativity and bricolage refer to the ability to creatively recombine available resources under the condition of resource constraints in entrepreneurial activities. Creativity and bricolage enable individuals to integrate and recompose limited resources in time, generate novel and valuable solutions and grasp fleeting entrepreneurial opportunities to facilitate entrepreneurial behavior in a resource-constrained environment (Vera and Crossan, 2005; Kumar and Shukla, 2022). Creativity is commonly associated with creative and innovative ideas for starting a new business (Cai et al., 2020a; Murad et al., 2021; Wang et al., 2021a). Duckworth et al. (2016) found a direct and positive relationship between creativity and entrepreneurial intentions. Murad et al. (2021) suggested that creativity is suitable for considering entrepreneurship as a career option and essential for initiating the entrepreneurial process, which leads to the design of new products. Therefore, creative individuals are more inclined to launch their own firms. Moreover, the research of Liu and Zhou (2020) research shows that bricolage can maximize the value of resources and encourage individuals to seize business opportunities and participate in business activities. In the entrepreneurial processes, predetermined preparations do not always work well, so creativity and bricolage are particularly essential to sense entrepreneurial opportunities and increase entrepreneurial intention.

In addition, Duxbury (2014) argues that time pressure is another crucial element implicit in improvisation. With increasing market competition and the acceleration of technological innovation, individuals do not have enough time to conduct detailed market research and need to rely on their intuition to make decisions and implement them quickly. Duxbury (2014) as a result, given the enormous pressure, the ability to function in stressful environments is critical to capturing unpredictable opportunities and realizing entrepreneurial behavior.

Furthermore, spontaneity and persistence—another dimension of improvisation—represent individuals’ action orientation and determination to achieve goals and solve problems (Hmieleski and Corbett, 2006, 2008). This dimension emphasizes the simultaneous occurrence of composition and implementation; in the face of emergencies, the time interval between planning and execution is almost the same. Individuals who are high in this dimension tend to prefer action rather than analysis and are highly concerned with the problem at hand (Vera and Crossan, 2005). Spontaneity and persistence allow individuals to identify entrepreneurial opportunities, integrate existing resources and adhere to their targets through actions.

Accordingly, in the Chinese context, with high uncertainty brought about by rapid technological and market changes, improvisation allows individuals to perceive and respond to environmental changes and spontaneously and creatively recombine the resources at hand. Improvisational individuals are more inclined to shape and seize entrepreneurial opportunities to increase their entrepreneurial intention. Thus, we propose the following:

*H1*: Improvisation is positively related to entrepreneurial intention.

### Improvisation and entrepreneurial self-efficacy

According to social cognitive theory, information about people’s capabilities has an impact on self-efficacy. Individuals with the ability to cope in emergency circumstances undoubtedly have a high perception of efficacy (Bandura, 1977). Therefore, improvisation enhances the advantages of survival and improves entrepreneurial self-efficacy.

As an essential element of improvisation, creativity and bricolage may have an impact on entrepreneurial self-efficacy. The creative use of resources at hand is often related to problem solving (Hansen et al., 2011; Antoncic et al., 2018). In a challenging environment, people are often determined to use various methods of overcoming obstacles and solving problems (Zhou et al., 2012). As such, successful problem solving can improve self-perception, leading one to engage in more challenging behaviors and tasks. Biraglia and Kadile (2017) show that the ideas generated by individuals using creativity can foster their self-confidence to perform related activities in a specific field. Sun et al. (2020) argue that resource bricolage enables individuals to find undiscovered entrepreneurial opportunities, which increases their confidence in entrepreneurship when facing more substantial resource constraints. Therefore, individuals with high creativity and bricolage are more convinced that they have entrepreneurial self-efficacy.

In addition, improvisational capabilities are critical in pressure-filled and stressful environments. People constantly face a lot of pressure when starting a business, for example, time pressure, role conflicts, and coping with past failures (Duxbury, 2014; Wei et al., 2015; Schmutzler et al., 2019). Differing sources of pressure cause potential entrepreneurs to doubt their entrepreneurial abilities. Klassen et al. (2013) show that developing teachers’ capabilities in managing overall work stress builds self-efficacy. When faced with risk and uncertainty in entrepreneurship, if potential entrepreneurs can overcome tremendous pressures and develop positive attitudes, they will gain more confidence in starting a business.

The opportunities in the entrepreneurial process are always “written in water” and require that individuals with improvisational capabilities, such as spontaneity and persistence, seize them. Adomako et al. (2016) state that opportunities are often fleeting and cannot be easily predicted. Individuals are required to react spontaneously instead of preparing for unknown situations (Vera and Crossan, 2005). In addition, resilient individuals are more inclined to follow the entrepreneurial path they chose and take actions to achieve goals (Gompers et al., 2010). Therefore, individuals who can respond spontaneously and pursue their goals persistently may have a greater chance of success, increasing entrepreneurial confidence.

In China, entrepreneurial practices have undergone significant transformations due to emerging technology and market changes. When faced with new complex problems in entrepreneurship, most individuals lack the available methods for reference or imitation (Hmieleski and Corbett, 2008; Bresman, 2010), leading to doubt and anxiety about one’s entrepreneurial choices. Improvisation forms a new source of emotional security in entrepreneurship to creatively identify practical solutions even if someone has insufficient experience, particularly enhancing entrepreneurial confidence. Therefore, to enhance entrepreneurial self-efficacy, we need to emphasize the role of improvisation. Thus, we propose:

*H2*: Improvisation is positively related to entrepreneurial self-efficacy.

### The mediating role of entrepreneurial self-efficacy in the relationship between improvisation and entrepreneurial intention

In light of the entrepreneurial event model, entrepreneurial self-efficacy is a prerequisite for entrepreneurial intention and behavior (Esfandiar et al., 2019; Cai et al., 2020b; Antoncic et al., 2021; Rakib et al., 2022). The entrepreneurial event model posits that perceptions of feasibility are directly influenced by self-efficacy. Feasibility can increase the propensity to take entrepreneurial actions and contribute to the entrepreneurial process by identifying and recognizing credible new entrepreneurial opportunities (Bullough et al., 2014). Entrepreneurial self-efficacy helps individuals generate entrepreneurial intention under the premise of high feasibility. The mediating role of entrepreneurial self-efficacy mainly focuses on the relationship between personality, risk propensity and entrepreneurial intention (Mei et al., 2017; Gu et al., 2018). However, there is no detailed explanation of the impact of improvisation on entrepreneurial intention *via* entrepreneurial self-efficacy.

Individuals often need to assess the uncertain external environment and relevant tasks when realizing their entrepreneurial ideas in China (Yu et al., 2018). Improvisation precisely offers them the confidence and courage to cope with unpredictability, enhance subjective initiatives and develop more preferences to generate entrepreneurial ideas (Magni et al., 2009). The achievement brought about through improvisation is an essential manifestation of examining whether participating in entrepreneurship is suitable. Individuals with improvisational capabilities are more inclined to adopt heuristic thinking (Hmieleski and Corbett, 2006; Leybourne and Sadler-Smith, 2006; Biraglia and Kadile, 2017). This kind of thinking helps people creatively use the resources at hand to generate innovative ideas and solutions to problems under time pressure and resource shortages. The achievement due to improvisation allows individuals to believe they can play a role in the entrepreneurial process, effectively enhancing entrepreneurial confidence and better perceiving entrepreneurial self-efficacy. Strong entrepreneurial self-efficacy has a promoting effect on the perception of entrepreneurial feasibility and makes them believe that they are capable of playing the role of an entrepreneur to show a significant predisposition toward nurturing entrepreneurial intention. Based on the above theories and analysis, we suggest that entrepreneurial self-efficacy plays a mediating role between improvisation and entrepreneurial intention. Thus, we propose:

*H3*: The relationship between improvisation and entrepreneurial intention is mediated by entrepreneurial self-efficacy.

### Moderated-mediation effect of entrepreneurial policy support

According to contingency theory, the external environment is a vital factor guiding individual behavior. Specifically, in a favorable situation, it is easier for an individual to achieve his or her established goals (Harrison, 2018). In China, with the development of entrepreneurial policies, improvisation may be more effective for enhancing entrepreneurial intention by entrepreneurial self-efficacy. Currently, the Chinese government is vigorously developing infrastructure construction and providing government incubators and venture capital-guided funds, which have effectively lowered the threshold for entrepreneurship, provided better entrepreneurial resources for individuals, enabling them to better display improvisational capabilities, enhanced entrepreneurial confidence and the feasibility of entrepreneurship (Korsching et al., 2001; Lan et al., 2018). With the support of entrepreneurial policies, improvisational ability is more easily transformed into entrepreneurial intention through entrepreneurial self-efficacy. The entrepreneurial policy support positively moderates the indirect relationship between improvisation and entrepreneurial intention through entrepreneurial self-efficacy. Thus, we propose:

*H4*: Entrepreneurial policy support moderates the relationship between entrepreneurial self-efficacy and entrepreneurial intention.

## Materials and methods

### Sampling and data collection

Some scholars advocate that the study of entrepreneurial intention should be conducted in the early stages of individual development. For example, research on the entrepreneurial intention of university students who have not yet started their careers can obtain a forward-looking perspective that avoids retrospective bias (Carter et al., 2003). In addition, university students are a relatively homogeneous group, which can effectively reduce the influence of individual differences on the research results and help understand the formation mechanism of entrepreneurial intention (Malebana, 2017). Data for this study were obtained through a questionnaire-based survey instrument implemented in China. According to the 2018 China Mass Entrepreneurship Index Report released by the Innovation and Entrepreneurship Research Center of Southwest Jiaotong University, we divide the regions into upstream and downstream regions. The report has now become an index monitoring system to observe the basic trend and entrepreneurial performance of “mass entrepreneurship and innovation” in China. To ensure the validity and generality of our results, we collected data from July to September 2018 from these regions as our survey locations: upstream regions, such as Jiangsu Province, Guangdong Province, Shanghai and Beijing, and downstream regions, such as Jilin Province and the Inner Mongolia Autonomous Region. A total of 450 questionnaires were distributed randomly, and 330 were returned. After excluding invalid questionnaires (with incomplete or inconsistent answers), we retained 251 valid questionnaires. To test non-response bias, we compared the early and late responses based on the assumption that the opinions of the late responses represented the opinions of non-respondents (Armstrong and Overton, 1977). Concerning entrepreneurial intention, the results of the *t*-test yielded no statistically significant differences between the early and late responses. Therefore, non-response bias does not seem to be a concern.

### Questionnaire and measures

We developed a questionnaire based on the theoretical literature widely cited. The questionnaire was first written in English and was then translated into Chinese according to the standard method of back-translation. Subsequently, the Chinese version was translated back into English by a third party for comparison with the first English version. This process was repeated until the two versions showed little substantive differences. After the translation, we sent the questionnaire to three professors to review, and then we revised it based on their suggestions. Next, a pilot test was conducted with 50 university students until no new feedback was received; we revised the questionnaire further based on the pilot study. To ensure the accuracy of the data, respondents received proper training before taking the survey.

All items are measured using five-point Likert-type scales drawn from the literature. University students were asked to score these constructs according to their views on the items, measuring them on a scale of 1 (strongly disagree) to 5 (strongly agree). The selected items measuring improvisation were proposed by Hmieleski and Corbett (2006). After the pilot test, we found many items on the scale led to inaccurate measurement so we deleted those with vague wordings and combined those with high similarity, eventually resulting in 13 items. Improvisation includes three dimensions: (1) creativity and bricolage, (2) the ability to function and excel under pressure-filled and stressful environments and (3) spontaneity and persistence. In this research, we consider improvisation to be a combination of these elements. We aggregated all items evaluating dimensions of improvisation to measure it completely. The scale developed by Forbes (2005) was adapted for use in this study to be suitable for China’s national conditions. We selected a seven-item scale related to entrepreneurial self-efficacy after combining similar items from the same domain. The five-item scale measuring entrepreneurial intention was selected based on Liñán and Chen’s (2009) scale in the literature. According to the global entrepreneurship monitor (2006), the measurement scale of the entrepreneurial policy support was constructed using a four-item scale. The GEM (2006) report points out that governments have an important role in encouraging entrepreneurial activity. The creation of institutions conducive to entrepreneurial activity, such as respect and enforcement of the rules of law, legal and financial transparency and a fair, competitive environment, is the fundamental responsibility of government (Bosma and Harding, 2006). In addition to these general principles, the entrepreneurial policy support in our study is at the regional level instead of the national level. As each region is at a different stage of development and faces different opportunities, effective policies for entrepreneurship need to be tailored to the local context (He et al., 2019). Thus, the entrepreneurial policy support was assessed from four aspects: preferential tax policies, registration and approval procedures, consulting services and local policies and regulations. The coefficient alphas of all variables are above 0.90. These results suggest that the theoretical constructs exhibit high reliability. This study also includes controls for several variables that might affect the hypothesized relationships, including demographic variables, such as gender, grade and major, studied in past research (Zhang et al., 2014; Piperopoulos and Dimov, 2015). Gender is a dummy variable, with a value of “1” assigned for male and a “0” assigned for female. Major is a dummy variable, with engineering assigned a value of “1” and others assigned a “0,” and management is assigned a value of “1” and others are “0.” In addition, a lower fear of failure is conducive to increasing entrepreneurial activities (Schmutzler et al., 2019). Therefore, “fear of failure would prevent individuals from starting a new business” (Fearfail) is also set as a control variable. “Fear of failure” includes a dummy variable with “1” representing when the fear of failure prevents an individual from starting a new business and “0,” otherwise.

## Results

### Measurement model

Following guidelines from Anderson and Gerbing (1988), a measurement model must be tested before evaluating the conceptual model. Exploratory factor analysis (EFA) with SPSS 22.0 software was used to identify underlying constructs. Principal axis factoring was carried out, followed by varimax rotation with Kaiser Normalization. Only factors with eigenvalues of more than one have been retained. All factors with eigenvalues less than one were considered insignificant and hence dropped. A total of four factors with eigenvalues greater than one were identified, which cumulatively explain 65.825 percent of the total variance of the data, namely, improvisation (factor 1), entrepreneurial self-efficacy (factor 2), entrepreneurial intention (factor 3) and entrepreneurial policy support (factor 4). All items used in the constructs are presented in Table 1. We then used a confirmatory factor analysis (CFA) with AMOS 22.0 software involving these four constructs. The measurement model provides a good fit to the data: *χ*^2^(371) = 836.444, *p* < 0.001, *χ*^2^/df = 2.255, RMSEA = 0.071, SRMR = 0.050, CFI = 0.909, IFI = 0.910, TLI = 0.901. LO-HI intervals for RMSEA are 0.064–0.077 within the acceptable range (Schreiber et al., 2006). Compared with three-factor, two-factor and one-factor alternative models, the results in Table 2 show that the four-factor model fits well. In addition, all fit indicators meet the required standards. Therefore, these items were retained; the factor loadings are presented in Table 1. Straub (1989) proposes that 0.5 is the cutoff level of the factor loadings of selected measures. Typically, loadings of 0.5 or greater are considered significant (Terziovski, 2010; Gunawan and Huarng, 2015; Lioukas and Reuer, 2015; Peña Häufler et al., 2021; Song et al., 2021; Wang et al., 2021b). The loadings of all items are basically greater than 0.7 and exceed 0.5, which shows adequate convergent validity.

**Table 1:** Factor loadings, Cronbach’s alpha, AVE and CR.

Four Factors and Scale Items	Factor Loading	Cronbach’s Alpha	AVE	CR
Improvisation Indicate your level of agreement with the following statements		0.929	0.509	0.930
I serve as a good role model for creativity	0.741			
I demonstrate originality in my work	0.781			
I take risks in terms of producing new ideas in completing projects	0.790			
I think outside of the box	0.773			
I identify opportunities for new services/ products	0.775			
I find new uses for existing methods or equipment	0.750			
I identify ways in which resources can be recombined to produce novel products	0.817			
I perform better under time pressure	0.702			
I need pressure in order to focus	0.662			
I “think on my feet” when carrying out actions	0.755			
I respond to problems in a “spur of the moment” way	0.594			
I am a persistent person	0.534			
I don’t let past failures hinder future performance	0.522			
ESE To what extent do you agree with the following statements regarding your degree of certainty in your ability to perform entrepreneurial-related task		0.913	0.605	0.914
Conduct market analysis	0.746			
Develop new markets	0.819			
Develop new products and services	0.772			
Conduct strategic planning	0.828			
Reduce risk and uncertainty	0.809			
Take calculated risks	0.796			
Perform financial analysis	0.662			
EI Indicate your level of agreement with the following statements		0.934	0.740	0.934
My professional goal is to become an entrepreneur	0.849			
I will make every effort to start and run my own firm	0.886			
I am determined to create a firm in the future	0.906			
I have very seriously thought of starting a firm	0.797			
I have the firm intention to start a firm some day	0.860			
EPS Indicate your level of agreement with the following statements		0.901	0.695	0.901
The government provides many preferential tax policies for entrepreneurship	0.840			
The registration and approval procedures of enterprises are simplified and convenient	0.785			
The government provides many consulting services for entrepreneurship	0.860			
The local government performed well in normalizing the policies and laws related entrepreneurship	0.848			

Note: CR: Composite reliability; AVE: Average variance extracted.

Table 1 demonstrates the average variance extracted (AVE) and composite reliability (CR). All AVE values exceed the 0.5 threshold (Fornell and Larcker, 1981), and all CR values are greater than the 0.7 critical value (Bagozzi and Yi, 2012). Table 3 displays the descriptive statistics and correlations in this study. Consistent with the theoretical logic we proposed, improvisation is positively associated with entrepreneurial self-efficacy and entrepreneurial intention. Entrepreneurial self-efficacy is positively associated with entrepreneurial intention. We also calculated the square root of AVE for each construct as shown in the diagonal elements of Table 3. The results demonstrate that the square root of AVE is greater than the correlations in the corresponding rows and columns, indicating good discriminant validity (Fornell and Larcker, 1981).

Common method variance (CMV) is generated when all variables are simultaneously measured using a single instrument (Malhotra et al., 2017). To avoid CMV as much as possible, we adopt procedural and statistical controls. The procedural techniques include protecting respondents’ anonymity, placing the constructs in different sections and improving scale items to reduce ambiguity. Concerning statistical techniques, Harmon’s single-factor model was tested by applying a CFA to reveal that the model fit the data poorly: *χ*^2^(377) = 2559.496, *p* < 0.001, *χ*^2^/df = 6.789, RMSEA = 0.152, SRMR = 0.126, CFI = 0.574, IFI = 0.577, TLI = 0.542. It indicates that the single-factor model is unacceptable, and CMV is unlikely to affect the results of this study (Tables 1-3).

**Table 3:** Variables mean, standard deviation and correlations.

Factors	Mean	SD	1	2	3	4	5	6	7	8	9
Gender	1.460	0.499	1.000								
Grade	2.430	0.862	0.056	1.000							
Engineering	0.371	0.484	–0.275**	0.054	1.000						
Management	0.251	0.434	0.279**	–0.036	–0.444**	1.000					
Fearfail	1.310	0.462	–0.161*	–0.115	–0.045	0.033	1.000				
EPS	3.075	0.848	–0.017	0.033	0.032	–0.008	0.158*	0.834			
Improvisation	3.270	0.607	–0.167**	–0.102	–0.009	–0.035	0.260**	0.407**	0.713		
ESE	3.239	0.680	–0.214**	–0.095	–0.026	0.000	0.272**	0.246**	0.614**	0.778	
EI	2.943	0.875	–0.235**	–0.042	0.059	–0.059	0.182**	0.078	0.470**	0.646**	0.860

Note: n=251; *p ≤ 0.05, **p ≤ 0.01; ***p ≤ 0.001.

**Table 2:** Confirmatory factor analysis.

Model	χ^2^/d.f.	TLI	IFI	CFI	RMSEA	SRMR
4-factor model	2.255	0.901	0.910	0.909	0.071	0.050
3-factor model^1^	3.692	0.787	0.805	0.804	0.104	0.086
2-factor model^2^	5.369	0.654	0.681	0.680	0.132	0.106
1-factor model^3^	6.789	0.542	0.577	0.574	0.152	0.126

Note: n=251;

1 Combines improvisation and entrepreneurial self-efficacy into potential factors;

2 Combines improvisation and entrepreneurial self-efficacy and entrepreneurial intention into potential factors;

3 Combines all variables into one variable.

### Mediating effect testing

**Figure 2:**
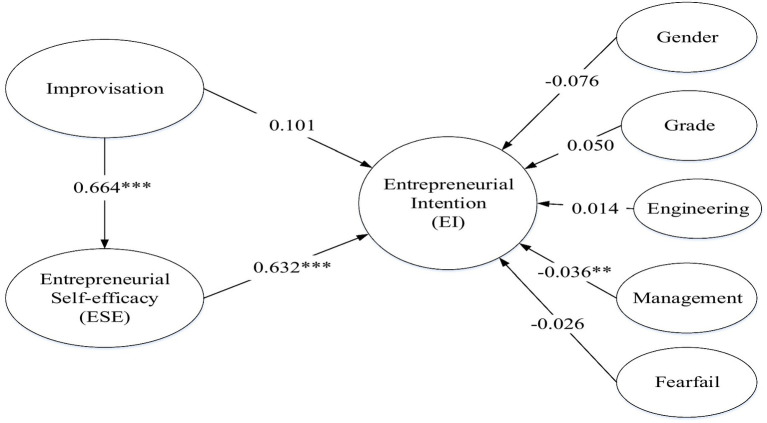
The analysis of mediation effect based on structural equation modeling.

After estimating the CFA model, we first used regression analysis with SPSS 22.0 software for evaluating H1 and H2, and then used structural equation modeling (SEM) with AMOS 22.0 software for evaluating the mediation analysis, not including the moderation effect. First, we found that H1 was supported by a regression analysis on the effect of improvisation on entrepreneurial intention (*β* = 0.434, *p* < 0.001). Second, we found that H2 was supported by a regression analysis on the effect of improvisation on entrepreneurial self-efficacy (*β* = 0.567, *p* < 0.001). Third, we adopted a SEM for testing H3. Figure 2 and Table 4 present the results of the SEM as well as the estimated effects, which provide a good model fit: *χ*^2^(382) = 836.584, *p* < 0.001, *χ*^2^/df = 2.190, RMSEA = 0.069, SRMR = 0.051, CFI = 0.901, and IFI = 0.902. As illustrated in the model, improvisation is positively and significantly related to entrepreneurial self-efficacy (*β* = 0.664, *p* < 0.001). Further, entrepreneurial self-efficacy is positively and significantly related to entrepreneurial intention (*β* = 0.632, *p* < 0.001). Improvisation does not have a significant impact on entrepreneurial intention (*β* = 0.101, *p* > 0.05). Therefore, entrepreneurial self-efficacy plays a fully mediating role between improvisation and entrepreneurial intention, supporting H3. In addition, this paper also used SPSS 22.0 software to examine whether there are differences in the results for different regions. The significance levels of different regions are basically the same, so the regions do not lead to significant differences in the formation of entrepreneurial intentions. In downstream regions (*N* = 136), improvisation is positively and significantly related to entrepreneurial intention (*β* = 0.434, *p* < 0.001) and entrepreneurial self-efficacy (*β* = 0.612, *p* < 0.001). Entrepreneurial self-efficacy plays a mediating role between improvisation and entrepreneurial intention (*β* = 0.566, *p* < 0.001). In upstream regions (*N* = 115), improvisation is positively and significantly related to entrepreneurial intention (*β* = 0.468, *p* < 0.001) and entrepreneurial self-efficacy (*β* = 0.482, *p* < 0.001). Entrepreneurial self-efficacy plays a mediating role between improvisation and entrepreneurial intention (*β* = 0.560, *p* < 0.001) (Table 4, Figure 2).

**Table 4:** Mediation model test.

Path	β	S.E	C.R	p
Gender⇒ EI	–0.076	0.075	–1.667	0.095
Grade⇒ EI	0.050	0.140	0.586	0.558
Engineering⇒ EI	0.014	0.096	0.293	0.769
Management⇒ EI	–0.036	0.050	–2.852	0.004
Fearfail⇒ EI	–0.026	0.163	–0.425	0.671
Improvisation⇒ ESE	0.664	0.075	8.824	0.000
ESE⇒ EI	0.632	0.114	7.572	0.000
Improvisation⇒ EI	0.101	0.103	1.327	0.184

Note: n=251; *β* = Coefficient estimates; S.E = Standard error; C.R = Critical ratio; *p* = Level of significance.

### Moderated mediation effect testing

After testing the mediation analysis, we used the bootstrap method with the SPSS process for evaluating H4. H4 suggests that entrepreneurial policy support interacts with entrepreneurial self-efficacy to impact entrepreneurial intention. A moderated mediation analysis is appropriate for testing the effects (Hayes, 2017). To test the moderated mediation model relationship provided four requirements without obtaining this moderated mediation do not exist. The suggestions are following, (a) the relationship between exogenous and endogenous should significant; (b) the interaction of moderator and mediator on endogenous should significant; (c) the relationship between the mediator and the endogenous variable should be significant; (d) the degree of conditional indirect effect has to be different at low, medium and high levels for moderator (Wang et al., 2021a). To test the conditional indirect effect through H4, Table 5 shows that (*β* = 0.234, *t* = 2.531, *p* < 0.05) significant relationship between improvisation and entrepreneurial intention and met with the condition (a). The interaction effect (*β* = 0.121, *t* = 2.111, *p* < 0.05) between entrepreneurial self-efficacy and entrepreneurial policy support is also significant that satisfies the condition (b). Table 3 shows that entrepreneurial self-efficacy has a direct positive and significant effect on entrepreneurial intention (*β* = 0.735, *t* = 9.347, *p* < 0.001) that met the condition criteria (c). Table 6 shows that the conditional indirect effect of improvisation on entrepreneurial intention through entrepreneurial self-efficacy (*β* = 0.838, *p* = 0.652; 1.025) that is positive and significant for high levels of entrepreneurial policy support (+1sd), and (*β* = 0.735, *p* = 0.580; 0.890) is also positive and significant for medium levels of entrepreneurial policy support (0) and (*β* = 0.633, *p* = 0.455; 0.811) is also a positive sign for low levels (−1sd) of entrepreneurial policy support but the degree of conditional indirect effect is different at low, medium and high levels for entrepreneurial policy support and accord with the condition (d). Thus, there is a conditional indirect effect of improvisation on entrepreneurial intention through entrepreneurial self-efficacy, supporting H4. We also found there is some differences in the level for entrepreneurial policy support. With low levels for entrepreneurial policy support, entrepreneurial self-efficacy has a significant positive effect on entrepreneurial intention, and with high levels of entrepreneurial policy support, although entrepreneurial self-efficacy also has a significant positive effect on entrepreneurial intention, and has more intense influence, indicating that with the increase of levels of entrepreneurial policy support, the effect of entrepreneurial self-efficacy on entrepreneurial intention is gradually increasing. In addition, at the three levels of entrepreneurial policy support, the mediating effect of entrepreneurial self-efficacy in the relationship between improvisation and entrepreneurial intention also showed an increasing trend. That is to say, with the improvement of the level of entrepreneurial policy support, the individual’s improvisational ability is more likely to enhance his entrepreneurial intention by improving entrepreneurial self-efficacy (Tables 5, 6).

**Table 6:** Conditional indirect effect of improvisation on entrepreneurial intention through entrepreneurial self-efficacy.

	β	S.E	Percentile 95% CI		p
			Lower Bound	Upper Bound	
The conditional indirect effect at high, medium and low entrepreneurial policy support					
Low (-1sd) entrepreneurial policy support	0.633	0.090	0.455	0.811	***
Medium (0) entrepreneurial policy support	0.735	0.079	0.580	0.890	***
High (+1sd) entrepreneurial policy support	0.838	0.095	0.652	1.025	***

Note: Bootstrapping sample size = 5000; β = Coefficient estimates; **p ≤ 0.05; **p ≤ 0.01; ***p ≤ 0.001.

**Table 5:** Direct, indirect and conditional effects.

Paths	β	S.E	t-Value	p	Bias-corrected Percentile 95% CI	
					Lower	Upper
X→Y	0.234	0.092	2.531	0.012	0.052	0.416
X→M	0.635	0.058	10.928	***	0.521	0.750
M→Y	0.735	0.079	9.347	***	0.580	0.890
M×W→Y	0.121	0.057	2.111	0.036	0.008	0.234
Controls						
Gender→Y	–0.103	0.092	–1.118	0.265	–0.284	0.078
Grade→Y	0.033	0.049	0.67	0.504	–0.063	0.129
Engineering→Y	–0.103	0.097	0.977	0.330	–0.097	0.287
Management→Y	–0.016	0.108	–0.15	0.881	–0.229	0.197
Fearfail→Y	0.012	0.095	0.129	0.897	–0.175	0.200

Note: n=251; X=Improvisation; M=Entrepreneurial self-efficacy; Y=Entrepreneurial intention; W=Entrepreneurial policy support; β = Coefficient Estimates; S.E=Standard error; *p* = Level of significance; Bootstrapping=5000; CI=Confidence of interval 95%; *p ≤ 0.05; **p ≤ 0.01; ***p ≤ 0.001.

## Discussion and implications

### Discussion

On the basis of social cognitive theory and the entrepreneurial event model, this article explores the influencing mechanism of improvisation on entrepreneurial intention in China’s transition economy and investigates the mediating role of entrepreneurial self-efficacy and the moderating role of entrepreneurial policy support in this relationship. Combining the theoretical research with the empirical study of data obtained *via* questionnaires, we find that improvisation has a positive effect on entrepreneurial intention and entrepreneurial self-efficacy and this relationship can be transmitted through the continuous mediating role of entrepreneurial self-efficacy and the moderating role of entrepreneurial policy support. Overall, this research initially improves the relationship between improvisation and entrepreneurial intention and introduces entrepreneurial self-efficacy and entrepreneurial policy support to open the “black box” in transitional economies, such as China, with high environmental uncertainty.

Concerning H1, it was predicted that improvisation is positively related to entrepreneurial intention, and this is accepted. Our empirical research results are parallel with Hmieleski and Corbett’s (2006) study of mature economies showing that improvisation has a positive relationship with entrepreneurial intention, which means that in both mature and transitional economies, improvisation can effectively promote entrepreneurial intention. When resource limitations are prohibitive and an individual is confronted with a novel entrepreneurial problem or opportunity, improvisation appears to be the most reasonable course of action. Individuals with a propensity for improvisation display a tendency toward self-selecting themselves into the field of entrepreneurship.

Regarding H2, it was predicted that improvisation significantly influences entrepreneurial self-efficacy, which is supported. Entrepreneurial self-efficacy has been recognized in relation to improvisation in terms of opportunity development, creativity and idea generation (Hmieleski and Corbett, 2008). This finding is similar to the recent studies of Balachandra (2019); which indicated that improvisation can promote an entrepreneurial mindset. The entrepreneurial mindset is founded on entrepreneurial self-efficacy, which is a broader definition of self-efficacy that encompasses the entire process of beginning a firm, allowing individuals to recognize their ability to adapt and/or act in crucial moments.

In H3, it was proposed that entrepreneurial self-efficacy has a mediating effect in the relationship between improvisation and entrepreneurial intention, which is accepted. This finding is similar to previous research (Mei et al., 2017; Gu et al., 2018; Cai et al., 2020b). People with high self-efficacy tend to have more entrepreneurial intentions. Individuals with high levels of improvisation tend to be more comfortable dealing with situations of uncertainty and risk and, in fact, perceive the objectively same situation as less risky than others. Consequently, they are more likely to anticipate experiencing less anxiety about an entrepreneurial opportunity, to perceive a greater sense of control over outcomes, and to judge the likelihood of receiving positive rewards as being greater, all of which are associated with higher levels of entrepreneurial self-efficacy.

Discussing H4, we found that entrepreneurial policy support moderates the mediated relationship between improvisation and entrepreneurial intention by entrepreneurial self-efficacy. This result is consistent with earlier studies (Schmutzler et al., 2019; Neneh, 2022). While most people will analyze whether they have the requisite skills to start a business before opting to do so, it is also well-known that an entrepreneurial career is fraught with risk and challenges. Given the dangers and uncertainty inherent in an entrepreneurial career, entrepreneurial policy support allows individuals to leverage their improvisational ability to form their entrepreneurial intentions. Such entrepreneurial policy support results in a supply of financial and instrumental assistance, which encourages the development of entrepreneurial intentions in the face of uncertainty.

### Theoretical implications

Our research contributes to the existing theoretical literature in several ways. First, we analyzed the relationship between improvisation and entrepreneurial intention in China and examined it empirically, contributing to developing improvisation research and the self-efficacy theory. Although literature that focuses on this relationship in mature economies exists (Hmieleski and Corbett, 2006, 2008), few scholars have examined whether improvisation could be a predictor of entrepreneurial intention in China’s transitional economy. Compared to mature economies, the external environment in transitional economies exhibits a high degree of uncertainty (Yu et al., 2020). The transition from a planned economy to a market driven one changes fundamental assumptions, criteria and decision making and represents a genuine transformation, which requires a fundamental paradigm shift and a mentality that thrives on chaos. Therefore, our findings help expand the application of improvisation research in the context of transitional economies and emerging economies using data from China. In addition, existing research mainly sheds light on the influence of antecedents, such as entrepreneurial passion, emotional intelligence and entrepreneurship education on entrepreneurial self-efficacy (Piperopoulos and Dimov, 2015; Cai et al., 2020b). However, we propose the concept of improvisation as the source of entrepreneurial self-efficacy in particular to enrich the research on self-efficacy theory further.

Second, this research helps open the black box of the influence of improvisation on entrepreneurial intention by integrating the entrepreneurial event model with social cognitive theory. Our results indicate that entrepreneurial self-efficacy has a fully mediating role in the relationship between improvisation and entrepreneurial intention. It is crucial to explore how individuals can benefit from improvisation to expand the research on the antecedents of entrepreneurial intention further (Hmieleski and Corbett, 2006). Currently, many scholars consider entrepreneurial self-efficacy as an important mediator in the study of entrepreneurial intention (Mei et al., 2017; Wu et al., 2019; Cai et al., 2020b; Edwards et al., 2022; Kumar and Shukla, 2022). Since the existing literature does not provide detailed information to explain the mechanism of improvisation on entrepreneurial intention, we introduce entrepreneurial self-efficacy based on previous research to reveal the potential connections and attempts to provide preliminary evidence theoretically. The results also confirm the mediating role of entrepreneurial self-efficacy and provide evidence that entrepreneurial self-efficacy, as a perception of self-efficacy crucial for entrepreneurial intention, is greatly facilitated by improvisation.

Finally, our research enriches the entrepreneurial intention literature by providing deeper insight into the conditions under which entrepreneurial self-efficacy has a more substantial effect on entrepreneurial intention in China. The moderation model results show that the entrepreneurial policy support moderates the relationship between entrepreneurial self-efficacy and entrepreneurial intention. As He et al. (2019) point out, since 2000, China has encouraged people to start businesses in the more impoverished western regions through tax incentives and financial development. Under the background of “Mass Entrepreneurship and Innovation,” the overall entrepreneurial environment is gradually improving. However, due to the different stages of development and various opportunities, the entrepreneurial policies in each region are not the same. The idea of entrepreneurship is associated with the process of evaluation, discovery, exploration, and recognition of opportunities (Cai et al., 2020b). As shown in the moderated mediation model, in regions with better entrepreneurial policy support, entrepreneurial self-efficacy is more likely to form entrepreneurial intention. Thus, to stimulate entrepreneurial ideas, the transition economies of China, Brazil and Mexico have launched special entrepreneurial incentives (Covarrubias and Schiavon, 2018; Grover Goswami et al., 2019). Therefore, our study enriches the research on entrepreneurial self-efficacy and entrepreneurial intention based on policy enactment and encourages relevant empirical examinations in different contexts.

### Practical implications

Our results also have implications for entrepreneurial practices in challenging business environments like transitional economies. First, we provide significant insight for individuals navigating the context of transitional economies, such as China. In the face of environmental uncertainty, we suggest that improvisation may be a key capability that helps promote higher entrepreneurial intention. Western studies have shown that improvisation can increase entrepreneurial intention (Hmieleski and Corbett, 2006, 2008), and our research also indicates that improvisation favors entrepreneurial intention in transitional economies. China is an important transitional economy experiencing institutional change from central planning to market competition (Cai et al., 2017). Given that countries undergoing economic transition share similar contexts, our research findings are applicable in China and other countries in transition. Considering that individuals face higher entrepreneurial uncertainty in transitional countries, the development of improvisational capability has important practical significance for their entrepreneurial intention. As such, we call on other transitional countries to focus on the research of improvisation, which is crucial to solving problems in the transition process. Second, it is suggested that individuals increase their improvisational capabilities, as the ability will indirectly transfer to entrepreneurial intention through entrepreneurial self-efficacy. The latter is considered a “vehicle” that is likely to lead to entrepreneurial intention, and it is recommended that individuals enhance their entrepreneurial confidence so they can more easily realize entrepreneurial ideas. Third, the government’s intervention, such as entrepreneurial policies, provides a favorable climate to promote employment and entrepreneurship. Given this, we examined the entrepreneurial policy support in which individuals are more likely to transform entrepreneurial self-efficacy into entrepreneurial behavior, which has important practical significance for today’s social and economic development, particularly in transitional economies.

## Limitations and future research

Although this study has certain implications, several limitations require further attention. First, our research only examines the link using data from China’s transition economy. We call for this research to be replicated and extended to other transition economies, since the issues discussed here are relevant to all former centrally planned economies undergoing transitions. In future research, data from other transitional countries, such as Eastern European countries including the former Soviet republics, could be collected to increase the validity of research conclusions. Moreover, the results might be different in mature market economies where external environmental conditions are more stable. Further studies could compare transitional economies and mature economies. Additionally, in our research, some items with high similarity and vagueness were omitted based on the context of China, but these items have content validity based on the literature, and thus could be included in future research in a different context to further this research. Finally, the research data were collected at one point in time. The evolution of the relationship between key variables was not captured. In addition to a cross-sectional research approach, future research could apply longitudinal research methods, such as long-term tracking surveys, to examine the interaction between variables over time, such as whether entrepreneurial self-efficacy and entrepreneurial intention has feedback on improvisation over time.

## Data availability statement

The data presented in this study are available upon request from the corresponding author.

## Author contributions

All authors listed have made a substantial, direct, and intellectual contribution to the work and approved it for publication.

## Funding

This research was funded by National Natural Science Foundation of China (Grant No. 72072069), Basic Scientific Research Funds of Heilongjiang Province, Heilongjiang University Special Fund (Grant No. 2020-KYYWF-0976; 2020-KYYWF-0981), Undergraduate Teaching Reform research project of Jilin University (Grant No. 2021XYB105).

## Conflict of interest

The authors declare that the research was conducted in the absence of any commercial or financial relationships that could be construed as a potential conflict of interest.

## Publisher’s note

All claims expressed in this article are solely those of the authors and do not necessarily represent those of their affiliated organizations, or those of the publisher, the editors and the reviewers. Any product that may be evaluated in this article, or claim that may be made by its manufacturer, is not guaranteed or endorsed by the publisher.
